# Sampling Strategies for *Diceraeus melacanthus* in Early Maize: A Decision-Support Framework

**DOI:** 10.3390/life16060982

**Published:** 2026-06-11

**Authors:** Luciano Mendes de Oliveira, Rodolfo Bianco, Adriano Thibes Hoshino, Maurício Ursi Ventura, Pablo Ricardo Nitsche, Ivan Bordin, Ayres de Oliveira Menezes Júnior, Humberto Godoy Androcioli

**Affiliations:** 1 Entomology Laboratory, Instituto de Desenvolvimento Rural do Paraná—IAPAR-EMATER, Highway Celso Garcia Cid, Kilometer 375—Conjunto Ernani Moura Lima II, Londrina 86047-902, PR, Brazil; lucianomendes.oliveira@idr.pr.gov.br (L.M.d.O.);; 2 Jandaia do Sul Campus (UFPR), Universidade Federal do Paraná, João Maximiano Street, 426, Jandaia do Sul 86900-000, PR, Brazil; 3 Agronomy Department, Universidade Estadual de Londrina (UEL), Highway Celso Garcia Cid, Kilometer 380, Londrina 86057-970, PR, Brazil; 4 Agrometeorological, Plant Physiology and Food Science Laboratory, Instituto de Desenvolvimento Rural do Paraná—IAPAR-EMATER, Highway Celso Garcia Cid, Kilometer 375—Conjunto Ernani Moura Lima II, Londrina 86047-902, PR, Brazil; 5 Phytotechnics Laboratory, Instituto de Desenvolvimento Rural do Paraná—IAPAR-EMATER, Highway Celso Garcia Cid, Kilometer 375—Conjunto Ernani Moura Lima II, Londrina 86047-902, PR, Brazil

**Keywords:** green-belly stink bug, *Zea mays*, contagious distribution, IPM, presence–absence, decision making

## Abstract

The green-belly stink bug (GBB), *Diceraeus melacanthus* (Dallas, 1851) (Heteroptera: Pentatomidae) is a key South American maize (*Zea mays* L.) pest, feeding on seedlings and causing physiological disorders. Understanding *D. melacanthus* population distribution and establishing sampling plans is essential to manage this species. Hence, the objective was to determine a distribution pattern, recommend a sampling unit size and develop sampling plans for the GBB, covering maize pre-sow period up to the maize V4 stage. Assessments were carried out in an experimental field and nine crop fields in northern Paraná State. In the experimental field, quadrants of n, 2n, 4n, 8n and 16n (n = 0.25 × 0.25 m^2^) were tested, thus determining an aggregated distribution with a recommended sampling unit size of 0.5 × 0.5 m^2^. After the nine crop field samplings, a negative binomial distribution was deemed fit to represent GBB in field conditions. Two sampling plans were developed, highlighted is the sequential presence–absence plan, which recommends a maximum of 60 sample points, and a minimum of 25, with at least six presences to make control decisions. For a more assertive sampling, divide the evaluated area into glebes with distinct natural characteristics and employ the sampling plan to each glebe. These sampling plans must be validated before IPM recommendation.

## 1. Introduction

The green-belly stink bug (GBB), *Diceraeus melacanthus* (Dallas, 1851) (Heteroptera: Pentatomidae), has emerged as a primary pest in Neotropical agricultural systems. In soybean [*Glycine max* (L.) Merril] cultivation, *D. melacanthus* belongs to a complex of stink bugs that cause direct damage to seeds and pods. However, its impact extends beyond the summer season; following the soybean harvest, these insects seek refuge under vegetative residue, subsequently attacking succeeding crops such as maize (*Zea mays* L.) or wheat (*Triticum aestivum* L.). In Poaceae, *D. melacanthus* preferentially feeds on the seedling stalks, causing systemic physiological disturbances that can lead to significant stand loss [1,2].

Given the severity of early-season damage, effective population monitoring is critical during the initial phenological stages of maize. While previous research has established a control threshold of 0.8 individuals/m^2^ [3,4], the practical implementation of sampling remains a significant challenge. The prevalence of no-till systems, characterized by heavy surface mulch and stubble, provides a complex habitat where *D. melacanthus* can easily evade detection. In these environments, the pest subsists on fallen grains, volunteer plants, and emerging sprouts [5,6].

Characterizing the spatial distribution of *D. melacanthus* and determining the optimal sampling unit size are fundamental steps for transitioning from empirical control measures to precision-based Integrated Pest Management (IPM). Because the cryptic behavior of this insect under crop residues makes absolute counting both labor-intensive and cost-prohibitive, more efficient alternatives are required. Presence–absence (binomial) sampling plans offer a promising solution by replacing time-consuming individual counts with a binary assessment of pest presence, thereby reducing operational time while maintaining statistical reliability [7].

This study aims to: (i) elucidate the spatial distribution patterns of *D. melacanthus* in maize–soybean succession systems; (ii) identify the optimal sampling unit size that balances precision with cost-effectiveness; and (iii) develop a practical sequential sampling strategy suitable for field-level decision-making.

## 2. Materials and Methods

*Diceraeus melacanthus* adults, for spatial distribution and sampling unit size studies, were obtained from laboratory rearing (T: 25 °C; RH 70 + 10%; 14:10 h L:D), using green-bean pods (*Phaseolus vulgaris* L.), soybean [*Glycine max* (L.) Merr.] and peanut (*Arachis hypogaea* L.) grains. Distribution and sampling size studies were established in an experimental field in Londrina, Paraná State, Brazil (23°21′56″ S; 51°10′05″ O; 580 m altitude) at Instituto de Desenvolvimento Rural do Paraná—IAPAR-EMATER (IDR-Paraná). Climatic classification, according to Köppen, is humid subtropical with hot summers (Cfa), with an average annual precipitation of 1608 mm and temperature of 21.1 °C [8]. The soil taxonomy is classified as Rhodic Ferrosol with a very clayey texture [9]. Sampling plans were developed on crop field farms up to 90 km from the experimental station with natural field infesting insects and under similar climate and soil classification. Microsoft Office Excel (Microsoft 365 Online) and BioStat (5.0 version) were used for all data curation and analysis [10].

The study was divided in two main assays. The first, which was with controlled field conditions, served to establish *D. melacanthus* spatial distribution and determine the best fit sampling size. The second assay was carried out throughout various maize cultivating areas in the Paraná state and served to construct the sampling plans based on the results from the first assay, but now applied to non-controlled field conditions. The greater variation in population dynamics sampled throughout the second assay enabled the robust data collection and enhanced mathematical precision during the calculations.

### 2.1. Spatial Distribution and Sampling Unit Size

#### 2.1.1. Assessment Concept

To validate the baseline accuracy, net relative cost (NRC) and net relative accuracy (NRA) of the different sampling unit sizes, a controlled assay composed of three plots (10 × 10 m^2^) were demarcated in a field portion (2 hectares), previously cultivated with soybean (summer) and maize (fall–winter). The entire area was carefully searched and no *D. melacanthus* was detected. Each plot was placed 40 m (center) from the other.

**Figure 1:**
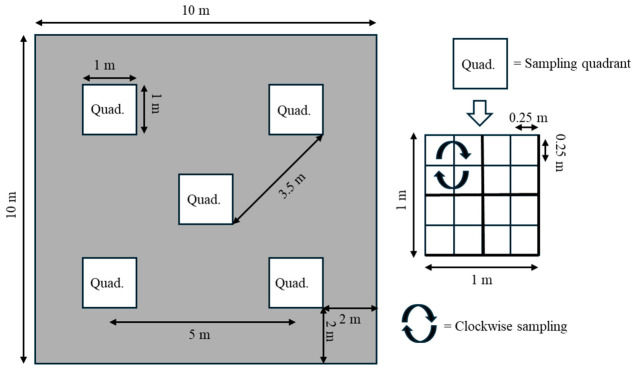
Experimental plan of each plot indicating the five evaluated quadrants with their respective subdivisions and spacing. Londrina, IDR-Paraná IAPAR-EMATER, 2026.

Inside each plot, five quadrants (1.0 m × 1.0 m^2^) were established in the borders (four) and in the center of the plot. Border quadrants were kept 2 m apart from the edges of the plot (Figure 1).

Each corner quadrant (midpoint) was 5 m apart and their distance from the central quadrant was 3.5 m (midpoint). Each quadrant was subdivided in four subplots made of aluminum square frames (0.25 m^2^, 50 mm thickness and 4 cm height); these frames were again internally subdivided in four equal experimental units (0.0625 m^2^) (Figure 1). The frames were made from a single aluminum beam [2.03 m (3 cm were consumed during the bending process)] which was bent to shape and each end of the beam was united with a smaller cut piece of aluminum and four rivets (two at each end of the beam); the internal division was made from two separate beams [52 cm; 2 cm were consumed during the bending to create a crease (1 cm) for two rivets attached on each side in contact with the frame] joint in the midpoint by making a vertical 25 mm indent on each beam; the entire construction is held together using a total of 12 rivets; the frame average weight was 580 g. Adult *D. melacanthus* (n = 400) with 10 days of adult maturity was released in the center of the experimental plot.

To reduce insect movement during assessment, quadrants were individually sprayed using a pyrethroid insecticide beta-cyfluthrin (20 g active ingredient/ha).

Sprayings were done at 24, 36 and 48 h, per respective plot, after the insects were released; this was accomplished to reduce bias and incentivize natural dispersion. The parcels were assessed six hours after the sprayings using appropriate individual protection equipment (IPE).

#### 2.1.2. Assessments

Each aluminum frame was assessed in a clockwise direction beginning in the left superior subplot, going from left to right in each aluminum frame (Figure 1). The number of *D. melacanthus* was recorded and the insects eliminated to avoid being recounted. Assessment total time in each quadrant size was also recorded.

Since the Morisita methodology was followed [11], a sequential quadrant size growth was performed, which resulted in 16 subplots of 0.0625 m^2^, eight of 0.125 m^2^, four of 0.25 m^2^, two of 0.5 m^2^ and one subplot of 1 m^2^. This resulted in 80 experimental units (0.25 × 0.25 m^2^) per plot, and 240 units in total.

#### 2.1.3. Data Analysis

The following formulas were used to analyze and evaluate the data collected during the first (controlled) assay; this resulted in the determination of the best fit dispersion pattern and sampling unit size (SU). The population dispersion index was calculated using

(1) $$DI=S^{2}/\mu$$

where DI = dispersion index, S^2^ = sample variance and μ = sample mean.

After defining the aggregate pattern, the Morisita formula was used to compare sampling size corresponding to the aggregated size of the population [11]:

(2) $$I\delta =n\left({\sum ix}^{2}\;-\sum x\right)/\left({\left(\sum x\right)}^{2}-\sum x\right)$$

where n = number of samples, ∑x = sum of individuals found and ${\sum ix}^{2}\;$= sum of the squared densities at which the individuals were found.

The Morisita index (Iδ) was calculated for a series of squares, formed from a source experimental sampling unit (SU) area (*a* = 0.0625 m^2^) and subsequent with 2 × *a*, 4 × *a*, 8 × *a* and finally 16 × *a*. Each subsequent square must have twice the area of the previous one. Next, the ratios were calculated: (Iδ *a*/Iδ 2 × *a*); (Iδ 2 × *a*/Iδ 4 × *a*); (Iδ 4 × *a*/Iδ 8 × *a*); (Iδ 8 × *a*/Iδ 16 × *a*); these ratios were plotted against their respective frame sizes (2 × *a*, 4 × *a*, 8 × *a* and 16 × *a*). Change in orientation of the plotted line (increasing or decreasing) indicates a sample size which best corresponds to the aggregate size.

The sum of every parcel, given their respective sampling size, was later shown as a comparison to their individual values.

Based on the above and using samples with an area of 1 m^2^, divided into 16 equal parts, the following sampling plans were tested per plot:

(a) 80 samples of 0.0625 m^2^;

(b) 40 samples of 0.125 m^2^;

(c) 20 samples of 0.25 m^2^;

(d) 10 samples of 0.5 m^2^;

(e) 5 samples of 1.0 m^2^.

Next, randomness deviation tests for the dispersion indices (3) and Morisita indices (4) were carried out through frequency comparisons of the Chi-square test.

(3) $$X^{2}calc=S^{2}/\mu \times (N-1)$$

(4) $$X^{2}calc=I\delta i=1Nxi-1+N-i=1Nxi$$

Net relative cost (NRC) (5) and net relative accuracy (NRA) (6) were also evaluated [12,13]:

(5) NRC=TC×RV

where TC = cost (time), and RV= relative variation, calculated from the ratio between standard error (Sx) and sample mean (μ). The NRA (6) is the inverse of NRC:

(6) $$NRA={NRC}^{-1}$$

The coefficients of Taylor’s Power Law were also determined [14]:

(7) $$S^{2}=a\mu^{b}$$

where *S*^2^ is the sampling variance, μ is the sampling mean, *a* is the coefficient which varies with sample unit size and *b* is the intrinsic coefficient representing the species’ spatial aggregation index.

After determining the ideal sample size, the negative binomial distribution index (k) was estimated, first using the moments method [15]:

(8) $$k=\;\mu^{2}/(S^{2}-\mu )$$

where k = negative binomial distribution index. Then, to achieve a near-exact value of k, the maximum-likelihood formula was used [16], in which the value of k obtained with the prior method (8) was used as a starting point:

(9) $$Log(N/n_{0}\;)=\;k\times {log}_{10}\;(1+\mu /k)$$

where N = total samples; $n_{0}$ = null samples.

After determining k, the necessary number of samples to obtain a precision that complies with the standard errors of 5, 10 and 20% of the sample mean was calculated using the following formula:

(10) $$n=\;\;(1/\mu +1/k)/E^{2}$$

where n = total samples; E = predetermined standard error in decimal (0.05, 0.10 and 0.20).

### 2.2. Sampling Plans

#### 2.2.1. Study Environment

The assessments were achieved in nine rural cultivating fields that were previously selected with a minimal of 10 ha and cultivating no-tillage soybean which would be followed by maize in succession. They were pre-surveyed for GBB presence, using beat-cloth during soybean grain-filling, direct no-tillage residue inspection, history of maize damage and *D. melacanthus* necessity of control records.

#### 2.2.2. Assessment Concept

Fields were divided in four glebes, where 20 sampling points (0.25 m^2^) were accomplished in each (80 total assessment points per survey). Assessments were achieved from pre-sowing to V4 maize stadium. Field glebes were established to represent different natural environment variations: close to forest reserve, neighboring farms, intense field inclination variation, near roads or bodies of water. Farmer decision and management was not influenced and dead *D. melacanthus* insects were also recorded during the sampling. Nymphs were tallied with adults. Eggs were found on no-tillage residue, spontaneous soybean and maize saplings, and weeds but where not tallied. The field evaluations occurred from 17 March 2023 to 19 April 2023, starting at the V2 stadium; maize damage score was also attributed for the plant adjacent to the sampled area [4].

#### 2.2.3. Data Analysis

Data collected in the field during the second assay were recorded for calculating sampling means, variances and dispersion index, following the previously mentioned formulas and methods used during the first assay (Formulas (1)–(10)). The value of the negative binomial distribution index (k) was then recalculated using the maximum-likelihood Formula (9). Next, the distribution probability was also calculated [17], and accomplished the goodness-of-fit test for theoretical frequency distributions using the total sampling values (2480 sampling points):

(11) $$P(0)={\left(1+\mu /k\right)}^{-k}$$

(12)  P(x)= (k+x−1)/x(μ/(μ+k))P(x−1)

where x = sampled insect frequencies (x = 1, 2, 3, …).

The goodness-of-fit test for theoretical frequency distributions was accomplished using the Chi-square test.

The minimum frequency was fixed at 1, and the degree of freedom (Df) associated with Chi-square was obtained using the following:

(13) $$Df=\;N_{c}-N_{p}-1$$

where $N_{c}$ = number of frequency distribution classes; $N_{p}$ = number of estimated sample parameters. The established significance value was of 5%.

Furthermore, the U coefficient from Evans (1953) [18] was calculated and compared to the original author’s standard error graph of U[SE(U)], which aids in verifying the negative binomial distribution fit:

(14) $$U=S^{2}-(\mu +\mu^{2}/k)$$

The SE(U) value must be corrected using the simple formula of 10 multiplied by the square root of total samples. If the calculated U value is significantly lower than the corrected SE(U) value, the negative binomial distribution can be considered a satisfactory model [16,18].

Upon concluding that the observed distribution fits the negative binomial distribution, the sampling plan could be constructed. A sequential presence–absence sampling plan (binomial distribution) was also constructed.

Before constructing sampling plans, the affinity between the sampled insect density (insects/sample) and its respective infested sample proportion (ISP) must be established; this affinity was represented in a graph with an adjusted negative exponential regression.

After establishing the affinity, the sample plan control limits (superior and inferior) were suited to the previously established value of affinity, performed using the formula from Nyrop and Binns [19], in which the adjusted control limit (m*) is determined; the value of m* is derived from the Z coefficient calculated previously with the ISP formula:

(15) $$ISP=1(1-e^{-Z})$$

(16) $${log}_{e}(-{log}_{e}\left(1-ISP\right)=Z\;$$

(17) $$\;Z=A+B(m^{\prime })$$

where ISP = infested sample proportion; e = Euler constant; Z = control threshold adjusting coefficient; A = linear coefficient; B = angular coefficient; m* = adjusted control threshold. These results are represented using figures and a table.

The sampling plans’ superior and inferior limits were constructed considering a ± 30% of the adjusted control threshold (m*), respectively, accomplished to reduce the Type 1 and Type 2 errors during decision-making. Thus, the decision lines S0 (inferior) and S1 (superior) were calculated using the following formula:

(18) $$S_{0}=h_{0}+bN$$

(19) $$\;S_{1}=h_{1}+bN$$

where $h_{0}$ e $h_{1}$ = are the linear coefficients for the respective limits; b = angular coefficient; N = number of sampling units.

The linear coefficients are calculated as follows:

(20) $$h_{0}=({log}_{e}\;\beta /(1-\alpha ))/({log}_{e}\;(p_{1}\times q_{0})/(p_{0}\times q_{1}))$$

(21) $$\;h_{1}=({log}_{e}(1-\beta )/\alpha )/({log}_{e}\;(p_{1}\times q_{0})/(p_{0}\times q_{1}))$$

where alpha and beta = decimal pre-established values of accepted error (0.10); p0 = ratio between the inferior control limit and k; p1 = ratio between the superior control limit and k; q0 = p0 + 1; q1 = p1 + 1.

The angular coefficient (b) for the negative binomial plan was calculated as follows:

(22) $$b=k\;({log}_{e}\;q_{1}/q_{0})/({log}_{e}\;(p_{1}\times q_{0})/(p_{0}\times q_{1}))$$

The operational curve of probability to accept the null hypothesis (OCP) and average number of samples needed to accept the null hypothesis (ANS) for the negative binomial sampling plan was calculated:

(23) $$OCp\;(\%)=(({\left(1-\beta )/\alpha \right)}^{x}-1)/(({\left(1-\beta )/\alpha \right)}^{x}-{\left(\beta /(1-\alpha )\right)}^{x})$$

(24)  ANS (n)=(COp (h0−h1)+h1)/(m−b)

(25) $$m=((k(m0+k))/({\left(m1+k\right)}^{x}-1))/((1-m1(m0+k))/(m0{\left(m1+k\right)}^{x}))$$

where x = arbitrary value used in both formulas [x ≠ 0 (+∞; −∞)]; b = angular coefficient; m0 = inferior control limit; m1 = superior control limit.

The sampling plan following a binomial distribution (presence–absence) was calculated as follows:

(26) $$h_{0}=({log}_{e}\;\beta /(1-\alpha ))/({log}_{e}\;(p_{1}\times q_{0})/(p_{0}\times q_{1}))$$

(27) $$\;h_{1}=({log}_{e}\;(1-\beta )/\alpha )/({log}_{e}\;(p_{1}\times q_{0})/(p_{0}\times q_{1}))$$

(28) $$\;b=({log}_{e}\;(q_{1}/q_{0}))/({log}_{e}\;(p_{1}\times q_{0})/(p_{0}\times q_{1}))$$

where h0 and h1 = linear coefficients; b = angular coefficient; p0 = inferior control limit; p1 = superior control limit; q0 = 1 + p0; q1 = 1 + p1.

The OCP (24) and MNS (25) were also calculated for the sequential presence–absence plan; the only variation to the formula is the calculation of the m coefficient:

(29) $$m=({\left(q1/q0\right)}^{x}-1)/({\left(p1/p0\right)}^{x}-{\left(q1/q0\right)}^{x})$$

## 3. Results

**Figure 2:**
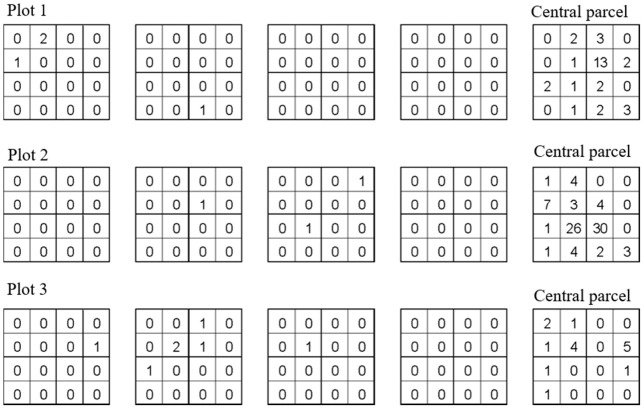
*Diceraeus melacanthus* sampled at the experimental field in the Instituto de Desenvolvimento Rural do Paraná—IAPAR-EMATER, in Londrina, Paraná State, (n = 400 stink bugs per plot) in five parcels subdivided in 16 equal parts (part area = 0.0625 m^2^). Londrina, Paraná State, IDR-Paraná, 2026.

Surveys conducted at the IDR-Paraná experimental field in Londrina, Paraná State, determined the theoretical spatial distribution of *D*. *melacanthus*. Total populations for each plot are summarized in Table 1 and Figure 2. The highest density was recorded in the second plot (36 h after release), where 89 stink bugs were sampled, representing 22.25% of the total population (N = 400). Conversely, the third plot (48 h after release) yielded the lowest density, with 23 insects (5.75%).

**Figure 3:**
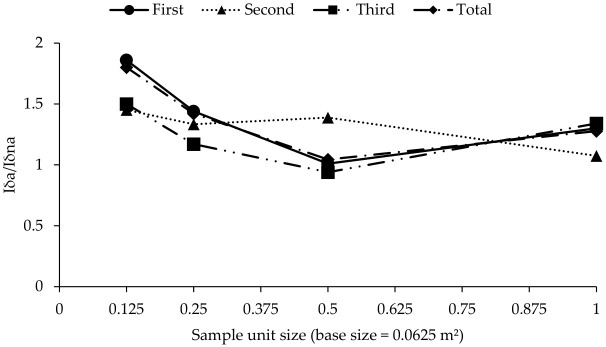
Morisita index (Iδ) ratios for their respective sample size in the first, second and third plot, considering *Diceraeus melacanthus* samples taken at an experimental field in the Instituto de Desenvolvimento Rural do Paraná—IAPAR-EMATER, in Londrina, Paraná State. 2026.

**Figure 4:**
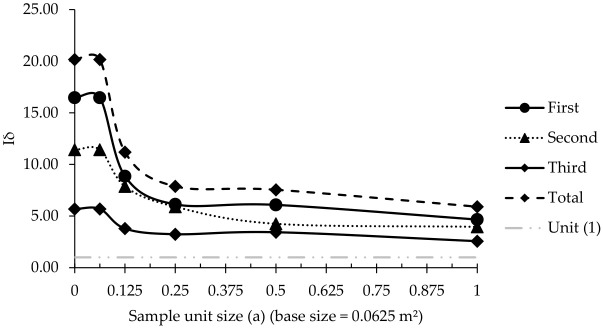
Morisita index (Iδ) for five sample sizes a, 2a, 4a, 8a and 16a (a = 0.0625 m^2^) in three evaluated plots and their bulk total, with the unit (1) line representing the standard, where random (Iδ = 1), normal (Iδ < 1) and aggregated (Iδ > 1) dispositions are seen. Londrina, Paraná State, IDR-Paraná, 2026.

The ratio between the Morisita index and respective sampling unit (SU) sizes indicated that 0.25 m^2^ (0.5 × 0.5 m^2^) and 0.5 m^2^ (0.5 × 1.0 m^2^) SUs best represent the *D*. *melacanthus* population (Figure 3). This selection is supported by the abrupt change in the slope of the plotted line for these specific SU ratios. Furthermore, individual Morisita index values were consistently greater than one, allowing the hypothesis of random or uniform distribution to be rejected in favor of an aggregated spatial distribution (Figure 4).

Evaluations of net relative cost (NRC), net relative accuracy (NRA), and relative variation (RV) are categorized by SU and plot (Table 2). The 0.25 m^2^ SU achieved the highest NRA with an average of 10 across all three plots, while the 1.0 m^2^ SU performed poorest with an average of 3. The 0.125 m^2^ SU (0.25 × 0.5 m^2^) followed with an NRA of approximately 9. Additionally, the 0.25 m^2^ SU exhibited the third-lowest sampling time (0.41–1.23 h). Given its superior NRA and competitive NRC, the 0.25 m^2^ SU was identified as the optimal sampling unit and was utilized for subsequent crop inspections.

Randomness deviation tests for the 0.25 m^2^ SU confirmed that the green-belly stink bug (GBB) follows an aggregate model (Table 3), a finding corroborated by the Morisita index analysis (Table 4).

The theoretical number of 0.25 m^2^ SUs required to estimate population density was calculated (Table 5). For a 10% standard error, the required samples ranged from 290 to 930; for 20%, the range was 50 to 230. While the ideal total was set at 3600 samples (45 evaluations across nine maize fields), logistical constraints limited the study to 31 evaluations (2480 sampling points). Thus, with a determined aggregated distribution (negative binomial fit) and most fit SU (0.25 m^2^), the study evolved into the second assay performed throughout nine areas in the Northen Paraná state.

**Figure 5:**
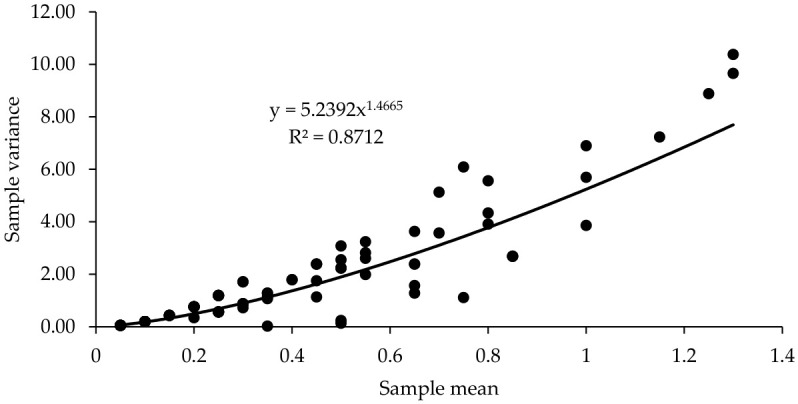
Taylor’s Power Law regression constructed using sample data from nine crop fields in the northern Paraná State, with a total of 117 composite samples made of 20 sample points each. Londrina, Paraná State, IDR-Paraná, 2026.

Taylor’s Power Law was calculated from data collected during the second assay and it yielded a “b coefficient” of 1.4665 with a high coefficient of determination (R^2^ = 0.87), further confirming an aggregated distribution (Figure 5). Consequently, the negative binomial distribution was solidly defined the most appropriate model to represent this species’ theoretical field distribution.

**Figure 6:**
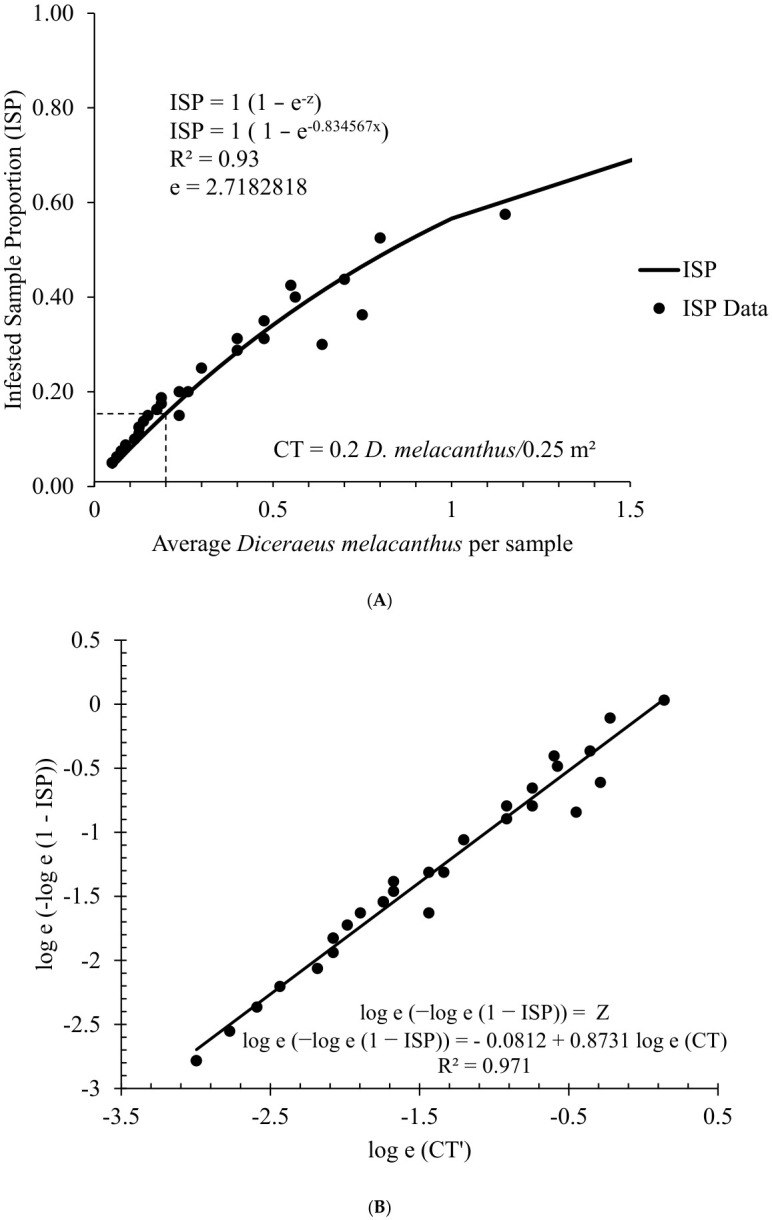
Negative exponential regression of the infested sample proportion (ISP) with the mean *Diceraeus melacanthus* per sample, superior (m1) and inferior (m0) thresholds (**A**). Empiric model adapted from Nyrop Binns (1992) to calculate the coefficient Z and adjust the control threshold (CT) by the infested sample proportion (ISP) [19]. (**B**) Londrina, Paraná State, IDR-Paraná, 2026.

Before constructing sequential sampling plans, the control threshold for *D*. *melacanthus* (0.80 GBB per m^2^) was adjusted for the 0.25 m^2^ SU, resulting in 0.20 GBB per 0.25 m^2^. Applying the infested sample proportion (ISP) formula as a final adjustment resulted in a threshold (m*) of 0.11 GBB per 0.25 m^2^, which was accomplished to fit the binomial distributions later used to construct the sampling plans (Figure 6A,B). Decision lines were established by adjusting this value by ±30%, resulting in an upper limit of 0.16 and a lower limit of 0.07 GBB per 0.25 m^2^ (Table 6).

**Figure 7:**
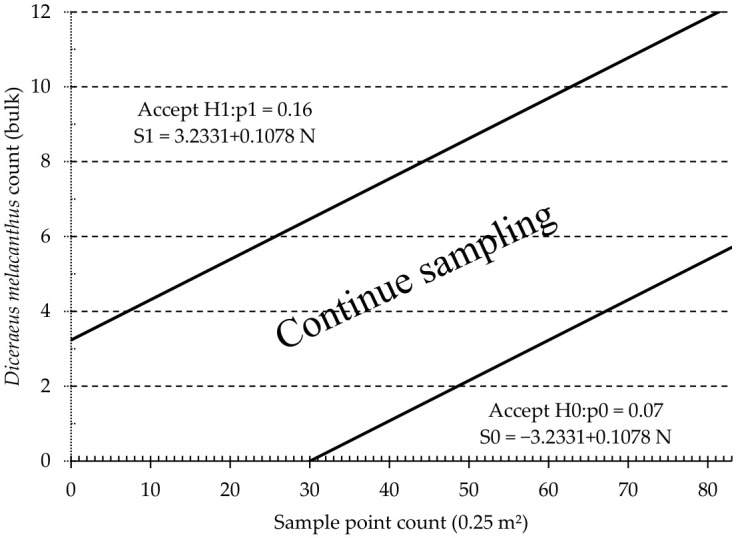
Sequential sampling plan constructed following a negative binomial distribution, including *D. melacanthus* count in bulk, sample count (N), with the alternate hypothesis (H1) of control decision, null hypothesis (H0) of no-control decision, upper control decision line (S1), lower no-control decision line (S0), upper threshold (p1) and lower threshold (p0) for Diceraeus melacanthus sampling. Londrina, Paraná State, IDR-Paraná, 2026.

**Figure 8:**
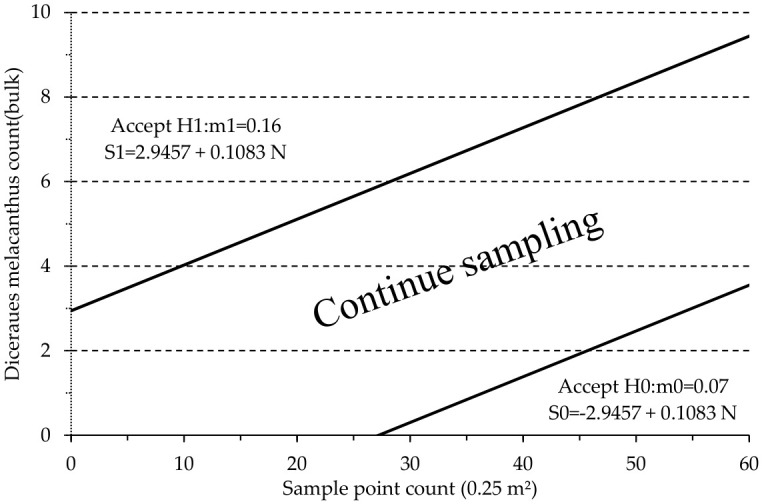
Sequential sampling plan constructed following a binomial distribution (presence–absence), including *D. melacanthus* presence in bulk, sample count (N), with the alternate hypothesis (H1) of control decision, null hypothesis (H0) of no-control decision, upper control decision line (S1), lower no-control decision line (S0), upper threshold (m1) and lower threshold (m0) for *Diceraeus melacanthus* sampling. Londrina, Paraná State, IDR-Paraná, 2024.

Chi-square tests (α = 0.05) and Evans’ adherence test (U = 0.187 < SE(U) = 4.98) confirmed that field data adhered to the negative binomial distribution (Table 7). The calculated sample mean was 0.345 *D*. *melacanthus* per 0.25 m^2^, with a sample variance of 0.77 and a dispersion index (DI) of 2.23. Null samples accounted for 77% of the data; 17% contained a single individual, and 6% contained at least two insects. Two sampling plans were subsequently developed: Negative Binomial-Based Composite Sequential Sampling Plan (k = 0.498): this plan uses composite samples of four points each (Figure 7, Table 8). It recommends a maximum of 80 sampling points for decision-making, with a minimum of 28 null points required to reach a “no-control” decision. For field application, 7 to 20 composite samples (four samplings each) are recommended between maize pre-seeding and the V4 stage. Binomial-Based Composite Sequential Presence–Absence Sampling Plan: this plan also uses composite samples (four samplings each) but utilizes a target threshold without requiring individual insect counts (Figure 8, Table 9). It requires a maximum of 72 points for a decision and a minimum of 24 null points for no-control.

**Figure 9:**
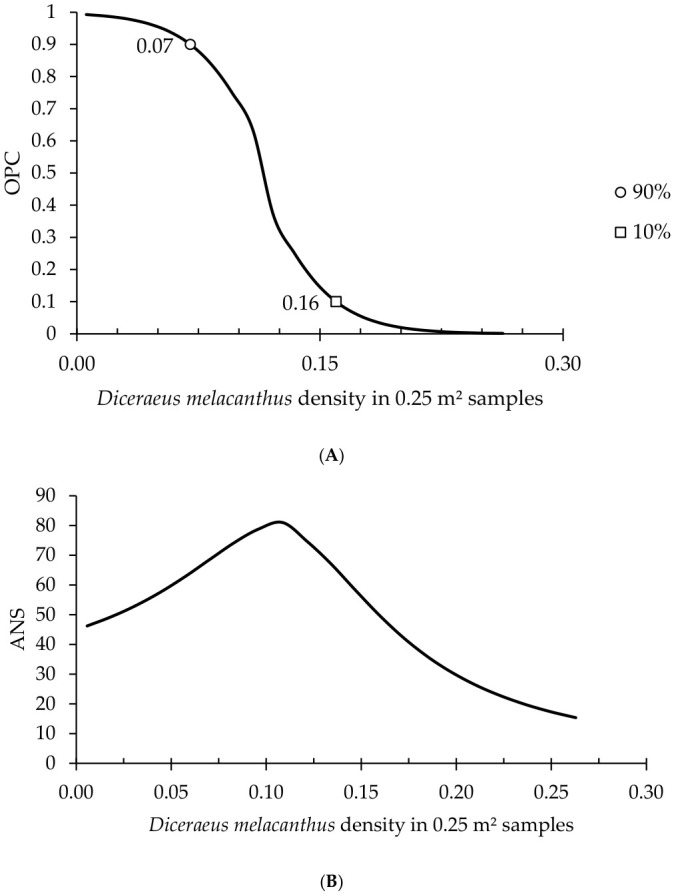
Operational curve for accepting the null hypothesis (OPC), with probabilities (90 and 10%) (**A**). The average number of samples (ANS) for *Diceraeus melacanthus* control decision-making (**B**). For the negative binomial distribution-based sampling plan of *D. melacanthus*. Londrina, Paraná State, IDR-Paraná, 2026.

**Figure 10:**
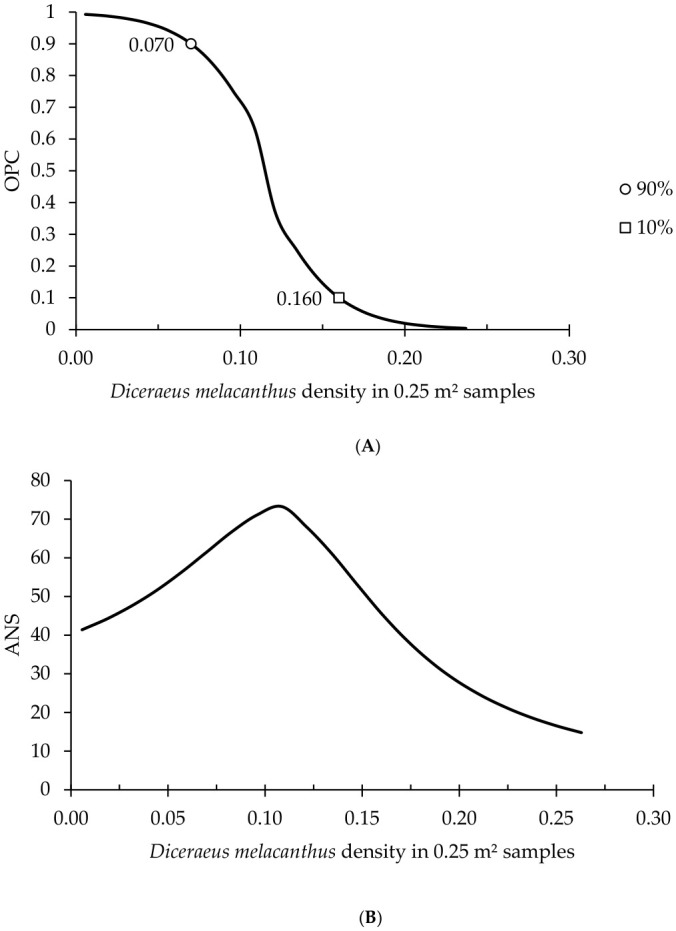
Operational curve for accepting the null hypothesis (OPC), with probabilities (90 and 10%) (**A**). The average number of samples (ANS) for *Diceraeus melacanthus* control decision-making (**B**). For the binomial distribution-based sampling plan of *D. melacanthus*. Londrina, Paraná State, IDR-Paraná, 2026.

The Operating Characteristic (OC) curves indicate a 90% probability of correctly accepting the null hypothesis at densities of 0.07 GBB (negative binomial-based plan) and 0.14 GBB (binomial-based plan) (Figure 9A and Figure 10A). The average number of samples (ANS) required reached its maximum at the 0.11 density threshold (73 and 81 points, respectively) and decreased significantly as pest density increased (Figure 9B and Figure 10B).

## 4. Discussion

The spatial distribution of *D. melacanthus* was determined by treating each quadrant (1 m^2^) as a representative of 1% of the plot area (Figure 1 and Figure 2). The consistent Morisita index values above 1 indicate significant population aggregation [11]. This gregarious behavior is characteristic of the adult stage, first-instar nymphs, and aggregated egg-laying patterns (masses up to 14 eggs). These findings align with previous studies documenting adult aggregate behavior in maize surveys [20].

The 0.25 m^2^ sampling unit was highlighted as the most fit representative size across all plots (Table 1, Figure 3). While the 0.5 m^2^ size also showed suitability based on the Morisita ratio [11], the 0.25 m^2^ unit provided the best balance between precision and labor, with a net relative precision (NRP) favoring a cost-effective 24 min assessment per 20 samples.

The adherence of field data to the negative binomial distribution was confirmed through multiple statistical lenses: the Chi-square test, Evans’s “U coefficient” [18], and Taylor’s Power Law [14]. The *b* coefficient of 1.4665 is consistent with values (± 1.5) typically associated with aggregated species [16]. These results corroborate the findings of Fernandes et al. (2022) for *D. melacanthus* and mirror distribution patterns observed in other Pentatomidae [20], such as *Nezara viridula* [21], *Euschistus heros* [22], and *Oebalus pugnax* [23].

While the sequential negative binomial plan (k = 0.498) is statistically proven, a sequential binomial presence–absence plan was also developed to increase field practicality. Presence–absence methods are often preferred in IPM (Integrated Pest Management) because they eliminate the need for time-consuming insect counts [24]. The high coefficient of determination (R^2^ = 0.93) for the infested sample proportion (ISP) relative to average density supports the validity of this approach (Figure 6A).

The Operating Characteristic (OC) curves and average number of samples (ANS) are vital for assessing the quality of these plans, particularly in minimizing Type II errors (failing to control a damaging population). Given that *D. melacanthus* causes the most significant damage between pre-seeding and the V4 stage [4,25,26] the sequential presence–absence plan offers a faster decision-making tool during this critical window.

The sequential presence–absence sampling plan demonstrates high potential for field application due to its relative operational simplicity. This approach differs from previous sampling plans for the green-bellied stink bug (GBB) and *Euschistus servus* (Say), a North American pentatomid pest of maize, which utilize plant clusters as sampling units [20,27,28]. Instead, this plan introduces a fixed-area evaluation method that enables rapid “no-control” decision-making, requiring a minimum of only 32 sampling points (equivalent to six composite samples). When implemented by trained professionals, the sequential presence–absence sampling plan minimizes assessment time and labor costs, thereby enhancing sampling efficiency and mitigating the damage potential of GBB. Consequently, this method optimizes decision-making and elevates the ecological and economic performance of IPM strategies. Conversely, the composite sequential sampling plan based on the negative binomial distribution is better suited for academic and research purposes, where precise pest quantification is required rather than binary presence–absence data.

To ensure maximum accuracy and efficiency when executing the composite sequential sampling plans in the field, a specific procedural protocol must be observed. First, the temporal window for monitoring *D*. *melacanthus* must initiate immediately following the soybean harvest. The sampling unit (SU) specification requires a fixed 0.25 m^2^ (0.5 × 0.5 m^2^) area, and utilizing a lightweight aluminum frame is highly recommended to facilitate transport and handling. For field stratification in heterogeneous fields with distinct landscape or soil characteristics, the acreage should be divided into uniform management zones (glebes), applying the sampling plan independently to each zone, though stratification is unnecessary in homogenous fields. To optimize spatial distribution prior to scouting, the sampling points should be mapped across the field or zone to ensure uniform coverage. Edge-effect mitigation is achieved by avoiding field borders, requiring a buffer zone where scouts move at least 5 m inward from the field edge before initiating data collection. Structurally, each composite sample comprises five individual SUs randomly distributed and evaluated across the selected area. To mitigate mechanical damage to vulnerable young plants during field scouting, the 0.25 m^2^ aluminum quadrant must be placed carefully over the seedlings rather than projected into the sampling area. To streamline the composite sampling process in the field, the four sampling units (SUs) can be arranged in an “X” configuration. Because each composite sample aggregates exactly to an area of 1 m^2^, this spatial arrangement aligns perfectly with standard Integrated Pest Management (IPM) practices, as most action thresholds for agricultural pests are inherently calibrated on a per-square-meter basis.

Implementing these standardized sampling protocols offers a path toward more sustainable Integrated Pest Management (IPM). By shifting from scheduled spraying to data-driven interventions, growers can improve control efficiency while reducing the economic and environmental costs associated with over-application of pesticides. Future research should focus on validating these models across broader geographical regions, beyond Brazil, such as Paraguay and Venezuela, to further refine the distribution coefficient (k) and reduce the environmental burden of preventative pesticide applications.

## 5. Conclusions

*Diceraeus melacanthus* adults exhibit an aggregated spatial distribution in maize fields, a pattern conclusively supported by Morisita index values, Taylor’s Power Law, and the robust fit of the negative binomial distribution. Among the evaluated dimensions, the 0.25 m^2^ (0.5 × 0.5 m^2^) sampling unit provides the optimal balance between high net relative accuracy and low operational cost, establishing it as the most efficient size for field assessments.

For commercial agriculture, the binomial-based presence–absence sequential sampling plan emerges as the most feasible approach. This protocol enables rapid population assessments during the critical management window, extending from pre-seeding to the V4 phenological stage, and ensures that control measures are triggered only when the adjusted economic threshold of 0.11 green-belly stink bugs per 0.25 m^2^ is exceeded.

These sampling plans should be validated before widely recommended and implemented in cropping scenarios.

## Figures and Tables

**Table 1:** Morisita indexes (Iδ) and ratio between these indexes for *Diceraeus melacanthus* populations (n = number of samples) (x = sampled individuals) and HAR = hours after release. Londrina, Paraná State, IDR-Paraná, 2026.

Plot	Statistical Parameter	Sample Unit Amount (n) (a = 0.0625 m^2^)				
		a	2a	4a	8a	16a
First (24 HAR)	n	80	40	20	10	5
	∑x	36	36	36	36	36
	∑x2	216	284	408	572	1034
	(∑x)2	1296	1296	1296	1296	1296
	Iδ	11.43	7.87	5.90	4.25	3.96
	Iδa/Iδna	-	1.45	1.33	1.39	1.07
Second (36 HAR)	n	80	40	20	10	5
	∑x	89	89	89	89	89
	∑x2	1701	1823	2493	4853	7401
	(∑x)2	7921	7921	7921	7921	7921
	Iδ	16.47	8.86	6.14	6.08	4.67
	Iδa/Iδna	-	1.86	1.44	1.01	1.30
Third (48 HAR)	n	80	40	20	10	5
	∑x	23	23	23	23	23
	∑x2	59	71	105	197	283
	(∑x)2	529	529	529	529	529
	Iδ	5.69	3.79	3.24	3.44	2.57
	Iδa/Iδna	-	1.50	1.17	0.94	1.34
Total	n	240	120	60	30	15
	∑x	148	148	148	148	148
	∑x2	1976	2178	3006	5622	8718
	(∑x)2	21,904	21,904	21,904	21,904	21,904
	Iδ	20.17	11.20	7.88	7.55	5.91
	Iδa/Iδna	-	1.80	1.42	1.04	1.28

**Table 2:** Calculation of net relative accuracy (NRA), net relative cost (NRC) and relative variance (RV) to establish the optimal sample size for *Diceraeus melacanthus*, data from three individual plots and their bulk total. Londrina, Paraná State, IDR-Paraná, 2026.

Plot	Samples Size (m^2^)	Number of Samples	Sample Total Cost (Hour)	Sample Mean	Sample Variance	RV	NRC	NRA
First	0.0625	80	0.40	0.29	1.82	0.53	0.21	9.1
Second				0.45	4.33	0.52	0.21	9.3
Third				1.11	20.96	0.46	0.18	11.8
Total		240	1.20	0.62	11.74	0.36	0.43	6.5
First	0.125	40	0.41	0.58	3.89	0.54	0.22	8.3
Second				0.90	7.11	0.47	0.19	11.1
Third				2.23	41.35	0.46	0.19	11.6
Total		120	1.23	1.23	24.27	0.36	0.45	6.1
First	0.25	20	0.41	1.15	4.92	0.43	0.17	13.3
Second				1.80	16.45	0.50	0.20	9.7
Third				4.45	95.05	0.49	0.20	10.3
Total		60	1.22	2.47	41.91	0.34	0.41	7.1
First	0.5	10	0.42	2.30	13.15	0.50	0.21	9.6
Second				3.60	40.52	0.56	0.23	7.6
Third				8.90	390.27	0.70	0.29	4.8
Total		30	1.26	4.93	152.29	0.46	0.58	3.8
First	1	5	0.53	4.60	34.22	0.57	0.30	5.9
Second				7.20	180.74	0.84	0.44	2.7
Third				17.80	1295.78	0.90	0.48	2.3
Total		15	1.58	9.87	463.64	0.56	0.89	2.0

**Table 3:** Chi-square (X^2^) randomness deviation test for the dispersion index (DI) with a sample unit size (SU) of 0.25 m^2^, showing sample point numbers (N) and data from three individual plots and their bulk total. Londrina, Paraná State, IDR-Paraná, 2026.

Plot	SU (m^2^)	N	Sample Mean	Sample Variance	DI	X^2^ Calculated	X^2^ Standard (0.025) *	X^2^ Standard (0.975) **
First	0.25	20	1.15	4.92	4.27	81.21	8.91	32.85
Second			1.80	16.45	9.14	173.67		
Third			4.45	95.05	21.36	405.82		
Total		60	2.47	41.91	16.99	322.83		

Liberty degree = N − 1, * confidence level (α = 0.025), ** confidence level (α = 0.975).

**Table 4:** Chi-square (X^2^) randomness deviation test for the Morisita indexes (Iδ) with a sample unit size (SU) of 0.25 m^2^, showing sample point numbers (N) and data from three individual plots and their bulk total. Londrina, Paraná State, IDR-Paraná, 2026.

Plot	SU (m^2^)	N	Iδ	X^2^ Calculated	X^2^ Standard (0.05) *
First	0.25	20	3.24	68.30	10.117
Second			5.90	190.67	
Third			6.14	471.22	
Total		60	7.88	1030.65	

Liberty degree = N − 1, * confidence level (α = 0.05).

**Table 5:** Ideal number of sampling points (INS) for best *Diceraeus melacanthus* population estimation in field conditions, considering the negative binomial coefficient (k), means from three plots and their bulk total, and fixed standard error (0.05, 0.1 and 0.2) for sampling units (SU) of 0.25 m^2^. Londrina, Paraná State, IDR-Paraná, 2026.

Plot	SU (m^2^)	Sample Count	Sample Mean	k	INS (0.05)	INS (0.10)	INS (0.20)
First	0.25	20	1.15	0.50	1148	287	51
Second			1.80	0.13	3299	825	193
Third			4.45	0.11	3726	932	227
Total		60	2.47	0.16	2662	666	157

**Table 6:** *Diceraeus melacanthus* control threshold (CT) adjustment considering the sample unit size (SU) and infested sample proportion (ISP) used to develop the negative binomial and binomial distribution sampling plans. Londrina, Paraná State, IDR-Paraná, 2026.

SU	CT *	ISP	Coefficient Z	Adjusted CT
0.25 m^2^	0.26 (+30%)	0.20	0.217	0.16
	0.20	0.15	0.167	0.11
	0.14 (−30%)	0.11	0.117	0.07

* Original *Diceraeus melacanthus* control threshold for maize of 0.8 stink bug × m^−2^ [3].

**Table 7:** Chi-square (X^2^) test (α = 0.05) and Evans (1953) coefficient U test for negative binomial fit of *Diceraeus melacanthus* theoretical distribution in crop field conditions, OF = observed frequency, EF = expected frequency and LD = liberty degree [18]. Londrina, Paraná State, IDR-Paraná, 2026.

Insect Frequency	Sum of the Frequencies	OF	EF	Calculated X^2^	Standard X^2^ (LD = 13)	U	SE(U) *
0	1911	0.7702	0.7695	0.573	3.565	0.187	4.98
1	419	0.1690	0.1568				
2	96	0.0391	0.0480				
3	26	0.0105	0.0164				
4	13	0.0052	0.0059				
5	4	0.0016	0.0022				
6	4	0.0016	0.0008				
7	2	0.0008	0.0003				
8	2	0.0008	0.0001				
9	0	0.0000	0.0000				
10	0	0.0000	0.0000				
11	1	0.0004	0.0000				
12	0	0.0000	0.0000				
13	1	0.0004	0.0000				
14	1	0.0004	0.0000				
Total	2480	1	1	Sampling mean = 0.345		Sampling variance = 0.77	

* Standard error adjusted for 2480 total samples [18].

**Table 8:** *Diceraeus melacanthus* composite sequential sampling plan (four sample points) constructed adopting a negative binomial distribution, with the superior (m1) and inferior (m0) thresholds, superior (S1) and inferior (S0) decision lines, alternate hypothesis (H1) of control and negative hypothesis (H0) of no-control, negative binomial coefficient (k) and fixed acceptable errors (α and β). Londrina, Paraná State, IDR-Paraná, 2026.

Composite Samples	*Diceraeus melacanthus* Individual Count Decision-Making		
	No-Control	≤Continue Sampling≥	Control
1	-	-	4
2	-	-	4
3	-	-	5
4	-	-	5
5	-	-	5
6	-	-	6
7 *	0	1 and 5	6
8	0	1 and 6	7
9	1	2 and 6	7
10	1	2 and 7	8
11	1	2 and 7	8
12	2	3 and 7	8
13	2	3 and 8	9
14	3	4 and 8	9
15	3	4 and 9	10
16	4	5 and 9	10
17	4	5 and 10	11
18	4	5 and 10	11
19	5	6 and 10	11
20	5	6 and 11	12
S0 = −3.2331 + 0.1078 N	m0 = 0.07		α = 0.10
S1 = 3.2331 + 0.1078 N	m1 = 0.16	k = 0.498	β = 0.10

* Minimum samples for decision-making.

**Table 9:** *Diceraeus melacanthus* composite sequential sampling plan (four sample points) constructed adopting a binomial distribution (presence–absence), with the superior (m1) and inferior (m0) thresholds, superior (S1) and inferior (S0) decision lines, alternate hypothesis (H1) of control and negative hypothesis (H0) of no-control and fixed acceptable errors (α and β). Londrina, Paraná State, IDR-Paraná, 2026.

Composite Samples	*Diceraeus melacanthus* Presence Decision-Making		
	No-Control	≤Continue Sampling≥	Control
1	-	-	3
2	-	-	4
3	-	-	4
4	-	-	5
5	-	-	5
6 *	0	1 and 5	6
7	0	1 and 5	6
8	1	2 and 5	6
9	1	2 and 6	7
10	1	2 and 6	7
11	2	3 and 7	8
12	2	3 and 7	8
13	3	4 and 8	9
14	3	4 and 8	9
15	4	5 and 8	9
16	4	5 and 9	10
17	4	5 and 9	10
18	5	6 and 10	11
S0 = −2.9457 + 0.1083 N	m0 = 0.07		α = 0.10
S1 = 2.9457 + 0.1083 N	m1 = 0.16		β = 0.10

* Minimum samples for decision-making.

## Data Availability

The raw data supporting the conclusions of this article will be made available by the authors on request.
